# Serum Magnesium Concentration Is Inversely Associated with Albuminuria and Retinopathy among Patients with Diabetes

**DOI:** 10.1155/2016/1260141

**Published:** 2016-07-31

**Authors:** Jun Lu, Yuying Gu, Meixiang Guo, Peihong Chen, Hongtao Wang, Xuemei Yu

**Affiliations:** ^1^Department of Endocrinology and Metabolism, Shanghai Jiao Tong University Affiliated Sixth People's Hospital South Campus, 6600 Nanfeng Road, Shanghai 201499, China; ^2^Department of Mathematics, Shanghai Business School, 2271 West Zhongshan Road, Shanghai 200235, China

## Abstract

*Aim*. To investigate the association between serum magnesium levels and microvascular complications among patients with diabetes.* Methods*. Patients with diabetes were recruited between April 2012 and January 2015. All patients received an assay of serum magnesium concentration, were screened for 24 h albumin excretion rate, and underwent nonmydriatic fundus photography. Albuminuria and retinopathy were defined accordingly. A total of 3,100 patients with normal serum magnesium levels were included in this study.* Results*. Patients with albuminuria and/or retinopathy had lower levels of serum magnesium than patients without these complications (*P* < 0.001). The prevalence of isolated albuminuria, isolated retinopathy, and combined albuminuria and retinopathy decreased as the concentration of serum magnesium increased. Multiple logistic regression analysis indicated that the odds ratio for isolated albuminuria, isolated retinopathy, and concomitant albuminuria and retinopathy decreased by approximately 20% for every 0.1 mmol/L increase in serum magnesium concentration.* Conclusion*. Serum magnesium levels were negatively associated with the risk of diabetic microvascular complications among patients with serum magnesium levels within the normal range.

## 1. Introduction

Magnesium is the fourth most abundant mineral in the body, is a cofactor for more than 300 enzymatic reactions, and is crucial for adenosine triphosphate (ATP) metabolism [1]. Magnesium is an essential mineral most notably present in foods rich in dietary fibre, nonstarchy vegetables, fruits, nuts, and dairy products [2]. Due to recent changes in eating habits, magnesium deficiency has become very common, especially for people with diabetes. Hypomagnesemia has been reported in 13.5% to 47.7% of nonhospitalized patients with type 2 diabetes compared to a prevalence of 2.5% to 15% in nonhospitalized patients without diabetes [3]. Low levels of magnesium have been associated with increased insulin resistance, the presence of type 2 diabetes mellitus, or even diabetes medication [4–6]. Dietary supplementation with magnesium may alleviate insulin resistance and decrease diabetes risk. In the Insulin Resistance Atherosclerosis Study, dietary magnesium intake was positively associated with increased insulin sensitivity after adjusting for confounding factors [7]. A meta-analysis provided further evidence that magnesium intake is significantly inversely associated with the risk of developing type 2 diabetes in a dose-dependent manner [8]. Supplementation with magnesium may also help control diabetes in patients with type 1 diabetes [9].

In addition to the correlation between hypomagnesemia and the risk of developing type 2 diabetes, hypomagnesemia is associated with chronic diabetic complications and increased mortality among critically ill patients with type 2 diabetes [10]. Wang et al. reported a negative association between serum magnesium levels and diabetic macrovascular complications, including cardiovascular disease and peripheral artery disease [5]. Serum magnesium depletion was also correlated with the presence of foot ulcers among subjects with type 2 diabetes [11]. However, the conclusions regarding the association between serum magnesium and diabetic microvascular complications are controversial. Hypomagnesemia may be correlated with diabetic retinopathy, microalbuminuria, clinical proteinuria, and neurologic abnormalities [12–14]. However, reports have also indicated no association between magnesium deficiency and diabetic microvascular complications [15].

Few large-sample studies exist regarding the correlation between magnesium depletion and diabetic microvascular complications in the Chinese population. We investigated the association between serum magnesium levels and microvascular complications among diabetic patients with normal serum magnesium levels.

## 2. Materials and Methods

### 2.1. Research Design and Study Population

**Figure 1:**
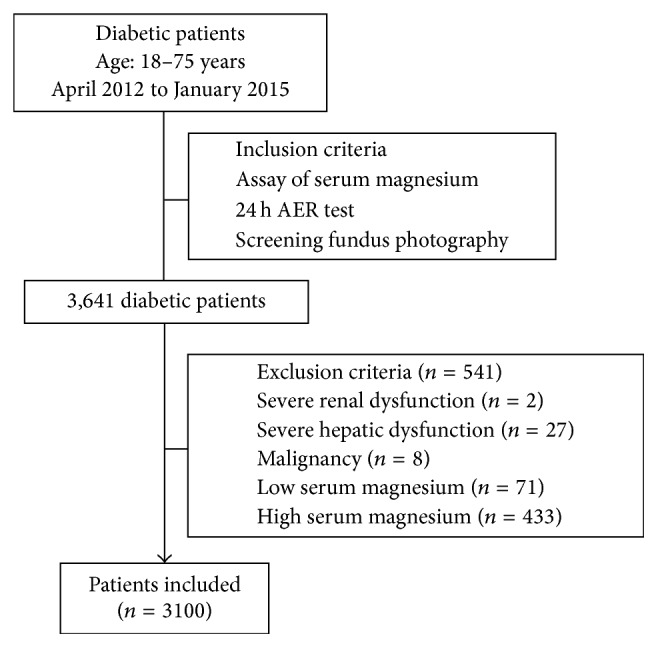
Flowchart of patient selection.

This study was conducted at Shanghai Jiao Tong University Affiliated Sixth People's Hospital South Campus, a tertiary hospital in Shanghai, China. This retrospective study evaluated chronic complications among patients with diabetes who were admitted to the Department of Endocrinology and Metabolism between April 2012 and January 2015. Patients with assay of serum magnesium, screening of microvascular complications including albuminuria and retinopathy, were included, and a total of 3,641 patients with diabetes aged 18–75 years were initially selected for this study. Patients with severe renal dysfunction (serum creatinine ≥ 450 *μ*mol/L, *n* = 2), severe hepatic dysfunction (alanine transaminase ≥ 260 U/L and/or aspartate amino transferase ≥ 220 U/L, *n* = 27), malignancy (*n* = 8), low serum magnesium level (*n* = 71), or high serum magnesium level (*n* = 433) were excluded from the study. Data regarding demographics, biochemical parameters, and microvascular complications were obtained from medical records. A total of 3,100 patients with normal serum magnesium levels (0.7–1.0 mmol/L) were included in this study (Figure 1).

The Shanghai Jiao Tong University Affiliated Sixth People's Hospital South Campus institutional review board approved this study in accordance with the principles of the Helsinki Declaration II. Informed consent was obtained from all patients included in the study.

### 2.2. Anthropometric and Biochemical Measurements

Height and weight were measured while the patients were barefoot and were wearing lightweight clothing. Body mass index (BMI) was calculated as weight divided by height squared (kg/m^2^).

Venous blood samples were obtained after at least 10 h of overnight fasting. Glycosylated haemoglobin (HbA1c) levels were measured using high-performance liquid chromatography (HLC-73G7, Tosoh, Tokyo, Japan). Plasma glucose levels were measured using the glucose oxidase method (Roche Diagnostics GmbH, Mannheim, Germany). Serum electrolytes and lipid profiles were determined with an autoanalyser (Hitachi 7600 analyser, Hitachi, Japan). Fasting C-peptide (FCP) was measured using electrochemical luminescence (Roche Diagnostics GmbH). The 24 h albumin excretion rate (24 h AER, mg/24 h) was measured on three consecutive days, and the average value was used for each patient.

### 2.3. Definition of Microvascular Complications of Diabetes

Diabetic retinopathy (DR) was graded according to the standards proposed by the American Academy of Ophthalmology (AAO, 2003) using nonmydriatic fundus photography [16]. Diabetic nephropathy (DN) was defined as a 24 h AER ≥ 30 mg/24 h.

### 2.4. Statistical Analyses

Data were expressed as medians (interquartile range, IQR) for non-normally distributed continuous variables or means ± standard deviations (SD) for normally distributed continuous variables. Categorical variables were expressed as numbers (%). Differences in means were calculated using one-way ANOVA with Dunnett analysis in regard to quantitative data and a Kruskal-Wallis *H* for non-normally distributed data; and differences in proportions were evaluated using the chi-square test. The association between serum magnesium levels and clinical characteristics was investigated with a Spearman correlation. The association of serum magnesium with albuminuria and retinopathy was assessed with a multivariable binary logistic regression. Tests for trends were performed using serum magnesium concentrations (0.1 mmol/L interval) as ordinal variables in the corresponding logistic regression models. All statistical analyses were performed using SPSS 19.0 (SPSS Inc., Chicago, IL, USA); two-sided *P* values < 0.05 were considered significant.

## 3. Results

### 3.1. Clinical Characteristics of Patients

The clinical characteristics of the included patients are listed in Table 1. A total of 3,100 patients with diabetes were included in this study. Patients with albuminuria and/or retinopathy had been diagnosed with diabetes for a longer duration and exhibited higher levels of systolic blood pressure and a higher prevalence of hypertension compared to patients without albuminuria and retinopathy (all *P* < 0.05). Patients with albuminuria and/or retinopathy also had significantly lower levels of serum magnesium than patients without the above complications (*P* < 0.05).

### 3.2. Frequency of Albuminuria and Retinopathy among Patients with Different Serum Magnesium Levels

**Figure 2:**
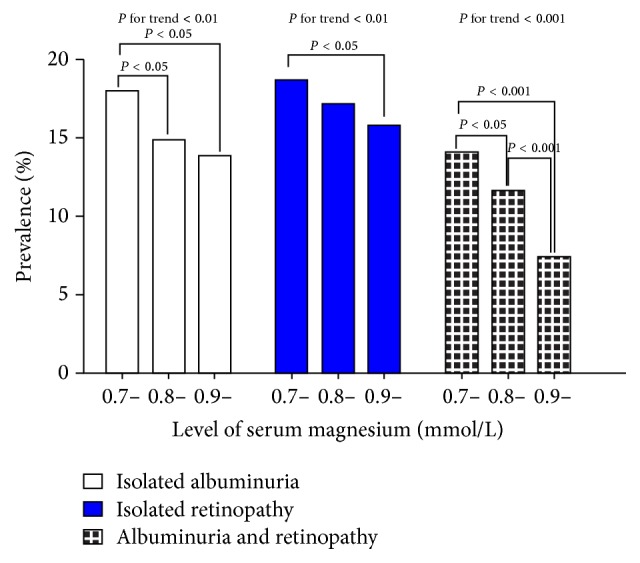
Prevalence of albuminuria and/or retinopathy among patients with diabetes stratified by serum magnesium levels. Data were analysed using the chi-square test.

As shown in Figure 2, the frequency of isolated albuminuria, isolated retinopathy, and concomitant albuminuria and retinopathy decreased as the level of serum magnesium increased (all *P* < 0.01). The binary logistic regression analysis indicated that the odds ratio of isolated albuminuria, isolated retinopathy, and concomitant albuminuria and retinopathy decreased by approximately 20% for every 0.1 mmol/L increase in serum magnesium (Table 2). Furthermore, patients in the highest tertile of serum magnesium levels had approximately a 30% to 60% decrease in the risk of isolated albuminuria, isolated retinopathy, and concomitant albuminuria and retinopathy compared with patients in the lowest tertile of serum magnesium levels after adjusting for confounding factors (*P* < 0.05).

### 3.3. Clinical Parameters Correlated with Serum Magnesium Levels

Serum magnesium levels were positively correlated with fasting and postprandial C-peptide and negatively correlated with levels of fasting plasma glucose (FPG), 120-minute postprandial glucose (120 min PPG), HbA1c, and C-reactive protein (CRP) (all *P* < 0.05). Other correlates of serum magnesium included gender and age (Table 3). No association was observed between serum magnesium levels and lipid profiles, blood pressure, BMI, or duration of diabetes.

## 4. Discussion

The association between magnesium and chronic microvascular complications among patients with diabetes has been investigated previously. However, inconsistent results were reported. Corsonello et al. pointed out that diabetic patients with microalbuminuria or clinical proteinuria exhibited a significant decrease in serum ionized magnesium concentration compared with patients who had normal levels of albumin in the urine [12]. Wang et al. reported an inverse correlation between serum magnesium levels and diabetic macrovascular complications [5]. A small study performed in China found no association between magnesium deficiency and microvascular complications [15]. In other studies, hypomagnesemia was associated with diabetic retinopathy among patients with diabetes [17, 18]. In the current study, the risk of isolated albuminuria, isolated retinopathy, and combined albuminuria and retinopathy decreased by approximately 20% for every 0.1 mmol/L increase in serum magnesium.

The mechanism behind the role of magnesium deficiency in the development of diabetic microvascular complications has not been well investigated. However, significant clues can be extrapolated from previous studies. Serum magnesium levels are associated with insulin resistance and *β* cell function in patients with diabetes. Furthermore, magnesium deficiency is associated with decreased *β* cell function and increased insulin resistance, leading to elevated plasma glucose levels [4–6]. Dietary supplementation with magnesium may decrease the risk of developing diabetes [7, 8, 19, 20]. However, magnesium supplementation and magnesium replacement do not improve insulin resistance in patients with metabolic syndrome [21]. In the current study, the serum magnesium levels were positively associated with fasting and postprandial C-peptide levels and negatively correlated with levels of fasting plasma glucose (FPG), 120 min PPG, and HbA1c (all *P* < 0.01). Additionally, low serum magnesium was related to increased proinflammatory and profibrogenic responses, which are risk factors for microvascular complications [22]. Serum magnesium levels are also negatively correlated with levels of CRP and IL-6 in patients with and without diabetes [23–26]. Oral magnesium supplementation decreased CRP levels in subjects with prediabetes and hypomagnesemia [27]. In the current study, serum magnesium was negatively associated with CRP levels (*r* = −0.045, *P* = 0.018). Additionally, low serum magnesium levels may promote endothelial cell dysfunction, reduce the activity of protective enzymes against oxidative stress, or interfere with DNA synthesis and repair [3]. The results from a low extracellular magnesium preparation for cultured endothelial cells demonstrated that maintaining magnesium homoeostasis might be a helpful and inexpensive intervention to prevent and treat endothelial cell dysfunction [28, 29].

The strengths of this study include a relatively large number of patients with diabetes for whom complete clinical data were recorded over the course of 3 years at a large general hospital; this is a relatively large sample size for research on chronic microvascular complications among patients with diabetes. However, our study also had some limitations. First, this was a retrospective study, and further prospective studies are required to investigate the association between magnesium deficiency and microvascular complications among patients with diabetes. Second, selection bias may exist because most patients included in this study were local residents of Shanghai, China. Thus, extrapolation of the conclusions of this study to other patient populations must be performed cautiously. Future and multicentre studies are warranted.

## 5. Conclusions

Although the patients had normal serum magnesium levels, serum magnesium concentration was inversely associated with diabetic microvascular complications. Supplementation of magnesium for diabetic patients may reduce the risk of diabetic microvascular complications.

## Figures and Tables

**Table 1:** Clinical characteristics of patients with and without albuminuria and diabetic retinopathy.

Variables	Absence of albuminuria and retinopathy (*n* = 1800)	Isolated albuminuria (*n* = 463)	Isolated retinopathy (*n* = 520)	Concomitance of albuminuria and retinopathy (*n* = 317)
Gender (male)	1064 (59.1)	306 (66.1)^*∗*^	285 (54.8)	185 (58.4)
Age (years)	55 ± 12	56 ± 12	56 ± 11	58 ± 10^*∗∗*^
Duration of diabetes (years)	4 (0.5–10)	5 (2–10)^*∗*^	8 (4–13)^*∗∗*^	11 (7–15)^*∗∗*^
BMI (kg/m^2^)	24.3 ± 3.7	25.9 ± 4.2^*∗∗*^	23.8 ± 3.5^*∗*^	25.2 ± 3.8^*∗∗*^
Blood pressure				
SBP (mmHg)	127 ± 16	135 ± 19^*∗∗*^	130 ± 17^*∗*^	142 ± 20^*∗∗*^
DBP (mmHg)	79 ± 10	83 ± 11^*∗∗*^	79 ± 10	84 ± 10^*∗∗*^
HTN (%)	750 (44.9)	281 (64.7)^*∗∗*^	236 (51.8)^*∗*^	226 (78.2)^*∗∗*^
HbA1c (%)	9.3 ± 2.5	9.2 ± 2.1	9.2 ± 2.1	9.6 ± 2.2^*∗*^
FPG (mmol/L)	8.6 ± 3.6	9.2 ± 4.4^*∗*^	8.8 ± 3.5	9.7 ± 4.3
120 min PPG (mmol/L)	13.9 ± 4.6	14.0 ± 4.6	14.1 ± 4.7	14.0 ± 5.2
FCP (ng/mL)	1.64 (0.91–2.39)	2.09 (1.24–2.99)^*∗∗*^	1.32 (0.77–2.01)^*∗*^	1.58 (0.84–2.37)
30 min CP (ng/mL)	2.30 (1.25–3.58)	2.80 (1.67–4.14)^*∗∗*^	1.80 (1.05–2.83)^*∗∗*^	1.97 (1.06–3.04)^*∗*^
120 min CP (ng/mL)	3.79 (1.89–5.72)	4.31 (2.36–5.96)^*∗*^	2.83 (1.52–4.81)^*∗∗*^	2.76 (1.53–4.7)^*∗∗*^
TC (mmol/L)	4.68 ± 1.10	4.83 ± 1.18^*∗*^	4.56 ± 1.06	5.13 ± 1.40^*∗∗*^
TG (mmol/L)	1.34 (0.93–2.00)	1.68 (1.16–2.59)^*∗∗*^	1.29 (0.86–1.88)	1.56 (1.05–2.24)^*∗*^
HDL-C (mmol/L)	1.15 ± 0.33	1.05 ± 0.28^*∗∗*^	1.18 ± 0.34^*∗*^	1.15 ± 0.33
LDL-C (mmol/L)	3.08 ± 0.94	3.08 ± 1.01	2.94 ± 0.90^*∗*^	3.41 ± 1.23^*∗*^
Serum Mg^2+^	0.88 ± 0.07	0.86 ± 0.07^*∗*^	0.87 ± 0.07^*∗*^	0.85 ± 0.07^*∗*^
CRP (mg/L)	1.0 (0.5–2.4)	1.6 (0.8–4.2)^*∗*^	0.9 (0.4–1.9)	1.3 (0.5–3.1)^*∗*^

Data are medians (interquartile range), means ± standard deviations, or numbers (%).

^*∗*^ *P* < 0.05, ^*∗∗*^ *P* < 0.001 comparison with subgroup of absence of albuminuria or retinopathy using one-way ANOVA with Dunnett analysis in regard to quantitative data and a Kruskal-Wallis *H* for non-normally distributed data.

BMI: body mass index, FPG: fasting plasma glucose, TC: total cholesterol, HTN: hypertension, TG: triglyceride, HDL-C: high-density lipoprotein cholesterol, LDL-C: low-density lipoprotein cholesterol, FCP: fasting C-peptide, 30 min CP: 30-minute postprandial C-peptide, 120 min CP: 120-minute postprandial C-peptide, SBP: systolic blood pressure, DBP: diastolic blood pressure, and CRP: C-reactive protein.

**Table 2:** Association of serum Mg^2+^ with albuminuria and retinopathy.

Mg^2+^ (mmol/L)	Isolated albuminuria		Isolated retinopathy		Albuminuria and retinopathy	
	OR (95% CI)		OR (95% CI)		OR (95% CI)	
	Model 1	Model 2	Model 1	Model 2	Model 1	Model 2
0.7–0.79	1	1	1	1	1	1
0.8–0.89	0.67 (0.49–0.90)^*∗*^	0.83 (0.58–1.19)	0.81 (0.60–1.09)	0.81 (0.57–1.15)	0.69 (0.49–0.99)^*∗*^	0.76 (0.49–1.17)
0.9–1.0	0.55 (0.41–0.75)^*∗∗*^	0.64 (0.44–0.92)^*∗*^	0.65 (0.48–0.88)^*∗*^	0.68 (0.47–0.97)^*∗*^	0.40 (0.27–0.58)^*∗∗*^	0.58 (0.37–0.91)^*∗*^
*P *value for trend	<0.001	0.009	0.004	0.030	<0.001	0.014

Per 0.1 mmol/L	0.76 (0.65–0.88)^*∗∗*^	0.79 (0.66–0.94)^*∗*^	0.81 (0.70–0.93)^*∗*^	0.83 (0.69–0.98)^*∗*^	0.62 (0.52–0.75)^*∗∗*^	0.76 (0.61–0.95)^*∗*^

Model 1: adjusted for gender, age, and duration of diabetes; Model 2: adjusted for gender, age, duration of diabetes, hypertension, BMI, HbA1c, TC, TG, and CRP.

Age, duration of diabetes, BMI, HbA1c, TC, TG, and CRP were analysed as continuous variables.

OR: odds ratio, 95% CI: 95% confidence interval, BMI: body mass index, TC: total cholesterol, TG: triglyceride, and CRP: C-reactive protein. ^*∗*^ *P* < 0.05; ^*∗∗*^ *P* < 0.001.

**Table 3:** Clinical characteristics correlated with serum Mg^2+^ (mmol/L).

Variables	Correlation coefficient	*P* value
Gender (male)	0.070	<0.001
Age (years)	0.106	<0.001
Duration (years)	−0.022	0.211
BMI (kg/m^2^)	−0.006	0.729
SBP (mmHg)	0.010	0.589
DBP (mmHg)	0.016	0.375
HbA1c (%)	−0.219	<0.001
FPG (mmol/L)	−0.135	<0.001
120 min PPG (mmol/L)	−0.122	<0.001
FCP (ng/mL)	0.056	0.002
30 min CP (ng/mL)	0.091	<0.001
120 min CP (ng/mL)	0.114	<0.001
TC (mmol/L)	0.004	0.819
Triglyceride (mmol/L)	−0.007	0.693
HDL-C (mmol/L)	0.020	0.273
LDL-C (mmol/L)	0.023	0.213
CRP (mg/L)	−0.045	0.018

Data were evaluated using Spearman correlation analyses.

SBP: systolic blood pressure, DBP: diastolic blood pressure, FCP: fasting C-peptide, 30 min CP: 30-minute postprandial C-peptide, 120 min CP: 120-minute postprandial C-peptide, HbA1c: haemoglobin A1c, TC: total cholesterol, HDL-C: high-density lipoprotein cholesterol, and LDL-C: low-density lipoprotein cholesterol.

## References

[1] Gröber U., Schmidt J., Kisters K. (2015). Magnesium in prevention and therapy. *Nutrients*.

[2] Wark P. A., Lau R., Norat T., Kampman E. (2012). Magnesium intake and colorectal tumor risk: a case-control study and meta-analysis. *The American Journal of Clinical Nutrition*.

[3] Pham P.-C. T., Pham P.-M. T., Pham S. V., Miller J. M., Pham P.-T. T. (2007). Hypomagnesemia in patients with type 2 diabetes. *Clinical Journal of the American Society of Nephrology*.

[4] Bertinato J., Xiao C. W., Ratnayake W. M. N. (2015). Lower serum magnesium concentration is associated with diabetes, insulin resistance, and obesity in South Asian and white Canadian women but not men. *Food and Nutrition Research*.

[5] Wang S., Hou X., Liu Y. (2013). Serum electrolyte levels in relation to macrovascular complications in Chinese patients with diabetes mellitus. *Cardiovascular Diabetology*.

[6] Hyassat D., Al Sitri E., Batieha A., El-Khateeb M., Ajlouni K. (2014). Prevalence of hypomagnesaemia among obese type 2 diabetic patients attending the National Center for Diabetes, Endocrinology and Genetics (NCDEG). *International Journal of Endocrinology and Metabolism*.

[7] Ma B., Lawson A. B., Liese A. D., Bell R. A., Mayer-Davis E. J. (2006). Dairy, magnesium, and calcium intake in relation to insulin sensitivity: approaches to modeling a dose-dependent association. *American Journal of Epidemiology*.

[8] Dong J.-Y., Xun P., He K., Qin L.-Q. (2011). Magnesium intake and risk of type 2 diabetes meta-analysis of prospective cohort studies. *Diabetes Care*.

[9] Lin C.-C., Huang Y.-L. (2015). Chromium, zinc and magnesium status in type 1 diabetes. *Current Opinion in Clinical Nutrition and Metabolic Care*.

[10] Curiel-García J. A., Rodríguez-Morán M., Guerrero-Romero F. (2008). Hypomagnesemia and mortality in patients with type 2 diabetes. *Magnesium Research*.

[11] Rodríguez-Morán M., Guerrero-Romero F. (2001). Low serum magnesium levels and foot ulcers in subjects with type 2 diabetes. *Archives of Medical Research*.

[12] Corsonello A., Ientile R., Buemi M. (2000). Serum ionized magnesium levels in type 2 diabetic patients with microalbuminuria or clinical proteinuria. *American Journal of Nephrology*.

[13] Hatwal A., Gujral A. S., Bhatia R. P. S., Agrawal J. K., Bajpai H. S. (1989). Association of hypomagnesemia with diabetic retinopathy. *Acta Ophthalmologica*.

[14] Sales C. H., Pedrosa L. D. F. C. (2006). Magnesium and diabetes mellitus: their relation. *Clinical Nutrition*.

[15] Xu J., Xu W., Yao H., Sun W., Zhou Q., Cai L. (2013). Associations of serum and urinary magnesium with the pre-diabetes, diabetes and diabetic complications in the Chinese Northeast population. *PLoS ONE*.

[16] Wilkinson C. P., Ferris F. L., Klein R. E. (2003). Proposed international clinical diabetic retinopathy and diabetic macular edema disease severity scales. *Ophthalmology*.

[17] Hamdan H. Z., Nasser N. M., Adam A. M., Saleem M. A., Elamin M. I. (2015). Serum magnesium, iron and ferritin levels in patients with diabetic retinopathy attending Makkah Eye Complex, Khartoum, Sudan. *Biological Trace Element Research*.

[18] Sharma A., Dabla S., Agrawal R. P. (2007). Serum magnesium: an early predictor of course and complications of diabetes mellitus. *Journal of the Indian Medical Association*.

[19] Xu T., Chen G. C., Zhai L., Ke K. F. (2015). Nonlinear reduction in risk for type 2 diabetes by magnesium intake: an updated meta-analysis of prospective Cohort studies. *Biomedical and Environmental Sciences*.

[20] Schulze M. B., Schulz M., Heidemann C., Schienkiewitz A., Hoffmann K., Boeing H. (2007). Fiber and magnesium intake and incidence of type 2 diabetes: a prospective study and meta-analysis. *Archives of Internal Medicine*.

[21] Lima de Souza E Silva Mde L., Cruz T., Rodrigues L. E. (2014). Magnesium replacement does not improve insulin resistance in patients with metabolic syndrome: a 12-week randomized double-blind study. *Journal of Clinical Medicine Research*.

[22] Fujita T., Hemmi S., Kajiwara M. (2013). Complement-mediated chronic inflammation is associated with diabetic microvascular complication. *Diabetes/Metabolism Research and Reviews*.

[23] Ata M. A., Shaikh S. S., Iqbal T. (2015). Inverse correlation between serum c-reactive protein and magnesium levels in smokers and nonsmokers. *North American Journal of Medical Sciences*.

[24] Chen Q., Zhao M., Guo F. (2015). The reduction of circulating levels of IL-6 in pregnant women with preeclampsia by magnesium sulphate and nifedipine: in vitro evidence for potential mechanisms. *Placenta*.

[25] de Oliveira A. R. S., Cruz K. J. C., Morais J. B. S. (2015). Magnesium status and its relationship with C-reactive protein in obese women. *Biological Trace Element Research*.

[26] Nielsen F. H. (2014). Effects of magnesium depletion on inflammation in chronic disease. *Current Opinion in Clinical Nutrition and Metabolic Care*.

[27] Simental-Mendía L. E., Rodríguez-Morán M., Guerrero-Romero F. (2014). Oral magnesium supplementation decreases C-reactive protein levels in subjects with prediabetes and hypomagnesemia: a clinical randomized double-blind placebo-controlled trial. *Archives of Medical Research*.

[28] Maier J. A. M. (2012). Endothelial cells and magnesium: implications in atherosclerosis. *Clinical Science*.

[29] Van Laecke S., Vanholder R. (2011). Magnesium and vascular dysfunction in malignant hypertension. *Hypertension*.

